# Carvedilol-Afforded Protection against Daunorubicin-Induced Cardiomyopathic Rats *In Vivo*: Effects on Cardiac Fibrosis and Hypertrophy

**DOI:** 10.5402/2011/430549

**Published:** 2011-04-14

**Authors:** Wawaimuli Arozal, Flori R. Sari, Kenichi Watanabe, Somasundaram Arumugam, Punniyakoti T. Veeraveedu, Meilei Ma, Rajarajan A. Thandavarayan, Vijayakumar Sukumaran, Arun Prasath Lakshmanan, Yoshiyasu Kobayashi, Sayaka Mito, Vivian Soetikno, Kenji Suzuki

**Affiliations:** ^1^Department of Clinical Pharmacology, Faculty of Pharmaceutical Sciences, Niigata University of Pharmacy and Applied Life Sciences, 265-1 Higashijima Akiha-Ku, Niigata City 956-8603, Japan; ^2^Department of Pharmacology, Faculty of Medicine, University of Indonesia, Jakarta 10430, Indonesia; ^3^Department of Pharmacology, Faculty of Medicine and Health Sciences, Syarif Hidayatullah Jakarta, State Islamic University, South Jakarta 15412, Indonesia; ^4^Department of Gastroenterology and Hepatology, Graduate School of Medical and Dental Sciences, Niigata University, Niigata 951-8510, Japan

## Abstract

Anthracyclines, most powerful anticancer agents, suffer from their cardiotoxic effects, which may be due to the induction of oxidative stress. Carvedilol, a third-generation, nonselective *β*-adrenoreceptor antagonist, possesses both reactive oxygen species (ROS) scavenging and ROS suppressive effects. It showed protective effects against daunorubicin- (DNR-) induced cardiac toxicity by reducing oxidative stress and apoptosis. This study therefore was designed to examine the effects of carvedilol on DNR-induced cardiomyopathic rats, focused on the changes of left ventricular function, cardiac fibrosis, and hypertrophy. Carvedilol increased survival rate, prevented systolic and diastolic dysfunction, and attenuated myocardial fibrosis and hypertrophy. DNR alone treated rats showed upregulated myocardial expression of ANP, PKC-*α*, OPN, and TGF-*β*1 and downregulation of GATA-4 in comparison with control, and treatment with carvedilol significantly reversed these changes. The results of the present study add the available evidences on the cardioprotection by carvedilol when associated with anthracyclines and explain the mechanisms underlying the benefits of their coadministration.

## 1. Introduction

Today, anthracyclines (doxorubicin, daunorubicin (DNR), epirubicin, and idarubicin) are among the most powerful drugs used for the treatment of oncologic diseases both in childhood and adulthood [1]. Regrettably, their clinical use is limited by the occurrence of dose-related cardiotoxicity [2]. This toxicity has elicited a large number of studies aimed at understanding both the mechanisms involved and the possible method to circumvent it. 

Anthracycline cardiotoxicity has been traditionally associated with oxidative stress-induced injury with a catalytic involvement of free cellular iron [3]. For many years, it has been believed that reactive oxygen species- (ROS-) induced degenerative changes are the primary hallmarks of anthracycline cardiomyopathy [4, 5]. Pathological studies on experimental animal models and human endomyocardial biopsies have shown that anthracycline-induced cardiomyopathy is characterized by histological alterations consisting in multiple areas of interstitial fibrosis associated with the presence of cardiomyocytes with vacuolar degeneration or compensatory hypertrophy. Necrotic cardiomyocytes with cellular infiltration, stromal edema with myocardial fibers dissociation, and the presence of polymorphonuclear cells can also be observed [6–8].

Carvedilol, a third-generation, nonselective *β*-adrenoreceptor antagonist that also possesses *α*1-adrenergic blocking property, has been shown to provide greater benefit than traditional *β*-adrenoreceptor antagonists in chronic heart failure because of its antioxidant, antiapoptotic, antiinflammatory, and antifibrotic properties [9]. A number of preclinical studies have demonstrated that carvedilol possesses both ROS scavenging and ROS suppressive effects compared to other *β*-adrenoreceptor antagonists in various experimental models of cardiac dysfunction and ischemia reperfusion [10–12]. Moreover, we and other investigators have recently reported that carvedilol prevents the development of cardiac toxicity induced by anthracyclines in rats [13], in the isolated perfused rat heart [14], and in patients receiving anthracyclines [15]. In our previous report, carvedilol showed protective effects against DNR-induced cardiac toxicity by reducing oxidative stress and apoptosis [13]. Nevertheless, it remains to be determined whether carvedilol can reduce cardiac fibrosis and hypertrophy in DNR-induced cardiomyopathic rats by its antioxidative and antiapoptotic effects. This study therefore was designed to examine the effects of carvedilol on DNR-induced cardiomyopathic rats and focused on studying the changes of left ventricular function, cardiac fibrosis, and hypertrophy.

## 2. Materials and Methods

### 2.1. Drugs and Chemicals

Unless otherwise stated, all reagents were of analytical grade and purchased from Sigma-Aldrich (Tokyo, Japan). DNR was kindly donated by Meiji Seika Ltd (Tokyo, Japan). Carvedilol was donated by Daichi-Sankyo Pharmaceutical (Tokyo, Japan).

### 2.2. Animals and Medication

Eight-week-old male Sprague-Dawley rats were obtained from Charles River Japan Inc. (Kanagawa, Japan). On day 0, each animal received a single intravenous injection of DNR at a dose of 3 mg/kg (i.v.). The drug was administered in three equal injections at 48-hour intervals for a period of a week to achieve an accumulative dose of 9 mg/kg, which is well documented to achieve cardiotoxicity [13, 16]. Twenty-two DNR-treated rats were randomly divided into two groups and received oral administration of carvedilol (30 mg/kg/day; group Carv; *n* = 10) or vehicle (group DNR; *n* = 12). Age-matched rats were injected with corresponding volumes of 0.9% NaCl and used as a control (group C; *n* = 5). Administration of carvedilol was started on the same day as DNR administration and continued for 5 additional weeks after cessation of DNR administration (6 weeks total period). Throughout the study, all animals were cared for in accordance with the guidelines of our institute and the Guide for Care and Use of Laboratory Animals published by the US National Institutes of Health. 

### 2.3. Cardiac Function Measurements

After the end of the study period (6 weeks), the body weight (BW) was measured, and rats were anesthetized with 2% halothane in O_2_ and subjected to surgical procedures. Left ventricular (LV) function analysis was carried out using hemodynamic and echocardiography measurement. After the instrumentation, the concentration of halothane was reduced to 0.5% to record steady-state hemodynamic data. Hemodynamic parameters such as mean blood pressure (MBP), peak LV pressure (LVP), LV end-diastolic pressure (LVEDP), and the rate of intraventricular pressure rise and decline (±dP/dt) were recorded as previously described [17]. 

Two-dimensional echocardiographic studies were performed under 0.5% halothane anesthesia using an echocardiographic machine equipped with a 7.5-MHz transducer (SSD-5500; Aloka, Tokyo, Japan). M-mode tracings were recorded from the epicardial surface of the right ventricle; the short axis view of the left ventricle was recorded to measure the LV dimension in diastole (LVDd) and LV dimension in systole (LVDs). LV fractional shortening (FS) and ejection fraction (EF) were calculated and expressed as percentages. The study was performed in a blinded manner.

### 2.4. Histopathological Analysis

After the measurement of echocardiographic parameters, hearts were excised and weighed immediately (HW), and its ratio to BW (HW/BW) was calculated. Half of each heart was immediately snap-frozen in liquid nitrogen for subsequent protein extraction and enzymatic assays. The remaining excised hearts were cut into about 2-mm-thick transverse slices and fixed in 10% formalin. After being embedded in paraffin, several transverse sections were obtained from the ventricle and stained with hematoxylin and eosin (HE) and Azan-Mallory to demonstrate interstitial edema, vacuolization, degeneration of cardiac fibers, and hypertrophy and fibrosis in cardiac tissues, respectively. The area of fibrosis (blue color) was quantified with a color image analyzer (Mac Scope; Mitani Co., Fukui, Japan).

### 2.5. Protein Analysis by Western Blotting

Protein lysate was prepared from heart tissues as described previously [18]. The total protein concentration in samples was measured by the bicinchoninic acid method [19]. For the determination of protein levels of atrial natriuretic peptide (ANP), transcription factor GATA-4, protein kinase C-alpha (PKC-*α*), osteopontin (OPN), and tumor growth factor-*β*1 (TGF-*β*1) equal amounts of protein extracts (30 *μ*g) were separated by sodium dodecyl sulfate polyacrylamide gel electrophoresis (Bio-Rad, CA, USA) and transferred electrophoretically to nitrocellulose membranes. Membranes were blocked with 5% nonfat dry milk in Tris-buffered saline Tween (20 mM Tris, pH 7.6, 137 mM NaCl, and 0.1% Tween 20). All the antibodies were purchased from Santa Cruz Biotechnology Inc. (CA, USA) and used at a dilution of 1 : 1000. The membrane was incubated overnight at 4°C with the primary antibody, and the bound antibody was visualized using the respective horseradish peroxidase secondary antibodies (Santa Cruz Biotechnology Inc.) and chemiluminescence developing agents (Amersham Biosciences, Buckinghamshire, UK). The level of glyceraldehyde 3-phosphate dehydrogenase (GAPDH) was estimated in every sample to check for equal loading of samples. Films were scanned, and band densities were quantified with densitometric analysis using Scion Image program (Epson GT-X700, Tokyo, Japan). All values were normalized by setting the density of normal samples as 1.0. 

### 2.6. Statistical Analysis

Data are presented as mean ± SEM and were analyzed using one-way analysis of variance (ANOVA) followed by Tukey or Bonferroni methods for *post hoc* analysis and two-tailed *t*-test when appropriate. A value of *P* < .05 was considered statistically significant. For statistical analysis, GraphPad Prism 5 software (San Diego, CA, USA) was used.

## 3. Result

### 3.1. General Toxicity

The general appearance and mortality of animals were recorded during the time course of the study. In the control group, no premature mortality was observed, and BWs were significantly increased during the experiment as compared with the initial values (408 ± 1.6 versus 538 ± 6.6 g; *P* < .05). On the other hand, premature death of six out of twelve animals (50%) with the presence of significant decrease in the BW was observed in DNR-treated rats (430 ± 12 versus 379 ± 9 g; *P* < .05 beginning vs end of study). Furthermore, in prematurely dead animals, a necropsy examination revealed a massive hydrothorax, ascites, and gastrointestinal bleeding. In addition, in comparison with the control group, the HW/BW was found to be significantly increased in this group (2.9 ± 0.06 versus 2.3 ± 0.02 g/kg, resp., *P* < .05). 

The decrease in BW was also found in the treatment group as compared with the initial values (423 ± 7.9 versus 405 ± 21 g; beginning versus end). The premature death was less when compared with DNR group. Two of ten animals (20%) were died during the time course of the study. Although carvedilol treatment tended to decrease the HW/BW compared with that in group DNR, the effect did not attain statistical significance (2.4 ± 0.02 versus 2.9 ± 0.06 g/kg, *P* > .05).

### 3.2. Effect of Carvedilol on Myocardial Functions

LVEDP was significantly higher (10.7 ± 0.3 versus 7.5 ± 0.9 mmHg, *P* < .05), and LVP and ±dP/dt were significantly lower in group DNR than in control group (111 ± 7 versus 124.3 mmHg, *P* < .05; 4800 ± 345 versus 6813 ± 541 mmHg/s, *P* < .05; 4135 ± 365 versus 7290 ± 775 mmHg/s, *P* < .05, resp.), indicating systolic and diastolic dysfunction in DNR rats. Carvedilol treatment improved the myocardial dysfunction by significant reduction in LVEDP (8.2 ± 1.2 versus 10.7 ± 0.3 mmHg, *P* < .05) and elevation in the LVP and +dP/dt (120.5 ± 11 versus 111 ± 7 mmHg, *P* < .05; 6229 ± 581 versus 4800 ± 345 mmHg/s, *P* < .05, resp.) compared with those in group DNR.

Echocardiographic data revealed that LV systolic function, as assessed by FS and EF, was reduced significantly in group DNR compared with that in control group (29.1 ± 1.3 versus 42.8 ± 1.7%, *P* < .05; and 59.6 ± 1.4 versus 78.9 ± 1.8%, *P* < .05, respectively). The reductions in both FS and EF were significantly attenuated in group Carv (38.8 ± 3.5 versus 29.1 ± 1.3%, *P* < .05; 73.6 ± 4.4 versus 59.6 ± 1.4%, *P* < .05, resp.). In addition, LVDd and LVDs were also enlarged in group DNR compared to group control (6.7 ± 0.5 versus 8.03 ± 0.3 mm, *P* < .05; 5.3 ± 0.2 versus 4.4 ± 0.3 mm, *P* < .05, resp.). The increase in both LVDd and LVDs were significantly attenuated in group Carv (7.05 ± 0.4 versus 8.03 ± 0.3 mm, *P* < .05; 4.9 ± 0.5 versus 5.3 ± 0.2 mm, *P* < .05, resp.). 

### 3.3. Effect of Carvedilol on Cardiac Histopathology

**Figure 1:**
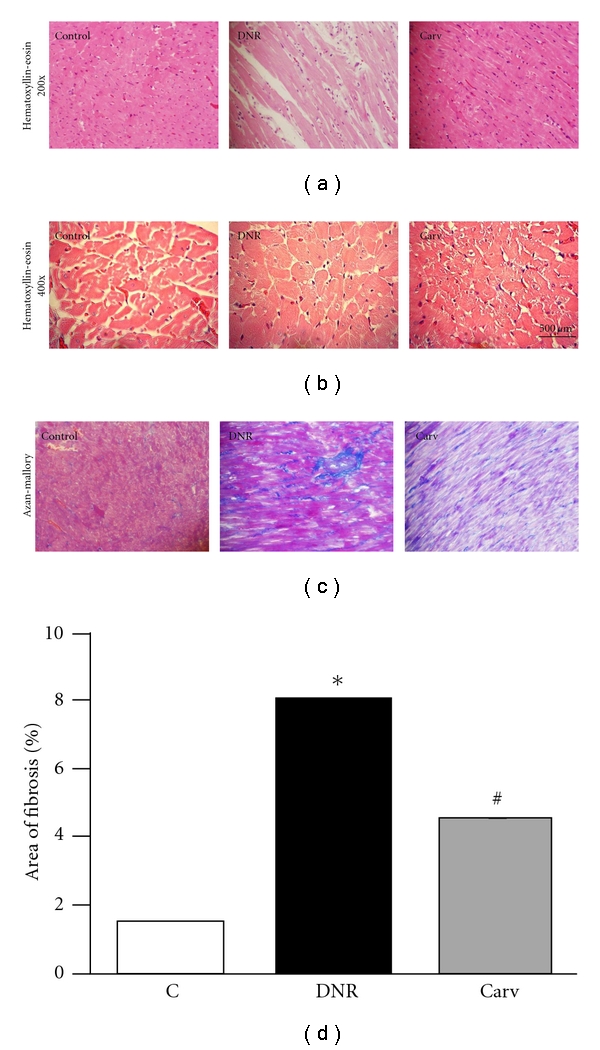
(a) Hematoxylin and eosin staining of the cross-sectional tissue slices of hearts depicting interstitial edema, vacuolization, and degeneration of cardiac fibers (X200). (b) Hematoxylin and eosin staining of the cross-sectional tissue slices of hearts depicting cardiac hypertrophy (X400), (c) Azan-Mallory staining for fibrosis of the cross-sectional tissue slices of hearts. Fibrosis is indicated by the blue area as opposed to the red myocardium (X200). (d) Bar graph showing % fibrosis in each experimental group. Each bar represents mean ± S.E.M. Group control (C) age-matched normal rats; group DNR, DNR-treated rats administered with vehicle; group Carv, DNR-treated rats administered with carvedilol (30 mg/kg/day). *P < .05 versus group Control; ^#^ *P* < .05 versus group DNR.

Histological changes in heart were evaluated, and the result is presented in Figure 1. Normal histology was seen in the control group (Figure 1(a)). On the other hand, there were several histological changes found in the DNR group (Figure 1(a)). Qualitatively, DNR-induced cardiac damage was recognized by the presence of marked interstitial edema, perinuclear vacuolization, disorganization, and degeneration of the myocardium. The damage in the form of lesions was rarely seen in the group treated with carvedilol compared with those in the DNR group (Figure 1(a)). 

Myocyte diameter was increased in group DNR compared with that in group C and reduced in group Carv (Figure 1(b)). Group DNR showed higher % of fibrosis (*P* < .05 versus group C); while in group Carv, fibrosis was significantly reduced (*P* < .05 versus group DNR) (Figures 1(c) and 1(d)).

### 3.4. Effect of Carvedilol on Myocardial Protein Expression of ANP, GATA-4, TGF-*β*1, PKC-*α*, and OPN Assessed by Western Blotting

**Figure 2:**
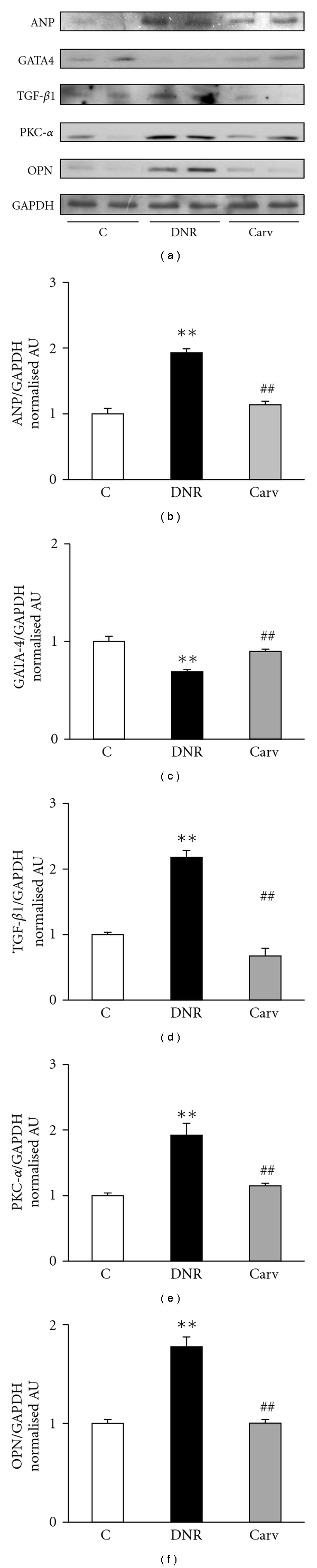
Myocardial expressions of ANP, GATA4, TGF-*β*1, PKC-*α*, and OPN. (a) Representative western blots showing specific bands for ANP, GATA4, TGF-*β*1, PKC-*α*, OPN, and GAPDH as an internal control. Equal amounts of protein sample (30 *μ*g) obtained from whole ventricular homogenate were applied in each lane. These bands are representative of five separate experiments. (b–f) Densitometric data of protein analysis. The mean density values of ANP, GATA4, TGF-*β*1, PKC-*α*, and OPN were expressed as ratios relative to that of GAPDH. Each bar represents mean ± S.E.M. Group control (C), age-matched normal rats; group DNR, DNR-treated rats administered with vehicle; group Carv, DNR-treated rats administered with carvedilol (30 mg/kg/day). **P < .01 versus group Control; ^##^ *P* < .01 versus group DNR.

Rats treated with DNR alone had an up-regulated expression of ANP, TGF-*β*1, PKC-*α*, and OPN in comparison with those in control group, and the treatment with carvedilol significantly reversed these changes (Figures 2(a), 2(b), 2(d), 2(e), and 2(f)). Moreover, myocardial protein expression of GATA-4 was decreased in group DNR compared with that in group Control, and carvedilol treatment significantly attenuated the decrease in GATA-4 (Figures 2(a) and 2(c)).

## 4. Discussion

In this study, repeated DNR administration resulted in increased mortality, which was accompanied by the symptoms of general toxicity. Hemodynamic and echocardiography measurements revealed a progressive decline in LV systolic and diastolic functions. Cotreatment with carvedilol was capable to protect the animals from cardiac dysfunction, and as a consequence the mortality rate was reduced. These findings illustrate the excellent cardioprotective potential of carvedilol, and the overall result is well in line with the outcomes of preclinical studies and clinical trials [13–15].

Cardiac hypertrophy, defined as an increase in cardiomyocyte size, is an adaptive response to a number of intrinsic (e.g., mutations of sarcomeric contractile proteins in familial hypertrophic cardiomyopathy) and extrinsic stimuli (e.g., hypertension). It is characterized by increased protein synthesis, sarcomeric reorganization, and re-expression of fetal regulatory genes. Prolonged pathological cardiac hypertrophy is a major cardiovascular endpoint and is strongly associated with arrhythmias, heart failure, and sudden death. In the present study, rats treated with DNR alone had developed cardiac hypertrophy and LV dilatation, which is evidenced by an increase in myocardial expression of ANP, that is documented to be elevated in cardiac hypertrophy or failure [20, 21], increase in myocyte size, HW/BW, LVDd, and LVDs, and decrease in FS and EF. Moreover, the myocardial expression of PKC-*α*, a gene related to myocardial hypertrophy [22], also increased in DNR rats. In the carvedilol-treated rats, a significant reduction of myocardial expression of ANP and PKC-*α*, myocyte size, HW/BW, LVDd, and LVDs and increase in the FS and EF has been observed. The above results indicated that carvedilol improves myocardial function and attenuates abnormal cardiac hypertrophy caused by DNR. To the best of our knowledge, this is the first study to perform the effect of carvedilol against cardiac hypertrophy induced by DNR. Anthracycline (doxorubicin) is thought to induce cardiac hypertrophy, a dose-limiting side effect, by the formation of free radicals and lipid peroxidation [23]. Previously, we have reported that DNR caused activation of NADPH oxidase subunits by increasing the myocardial levels of p47phox and p67phox, in addition to the increased malondialdehyde level and decreased glutathione peroxidase activity [13]. Carvedilol treatment significantly attenuated those changes [13]. Thus, we speculated that the ability of carvedilol to attenuate the cardiac hypertrophy caused by DNR in this study is through its ability to reduce the oxidative stress in DNR rats.

In the present study, the western blotting data showed that the DNR caused the significant reduction of myocardial expression of GATA-4, and cotreatment with carvedilol attenuated this reduction. The GATA-4 transcription factor is an important regulator of cardiac muscle cells [24, 25], and increased activities of GATA transcription factors could exert unwanted clinical manifestation such as cardiac hypertrophy [26]. Kim et al. [27] reported that anthracyclines can downregulate GATA-4 activity, and the mechanism of anthracyclines-induced cardiotoxicity may involve the downregulation of GATA-4 and the induction of apoptosis [27]. It is well established that carvedilol has antiapoptotic properties [28] and the inhibition of apoptosis as an important target for effective cardioprotection against antharcycline cardiotoxicity [29]. Recently, we have reported that DNR caused apoptosis by increasing the number of positive apoptotic cells, decreased myocardial level of Bcl-2, and increased myocardial level of caspase-7, and carvedilol treatment attenuated these changes [13]. Therefore, it is possible that the ability of carvedilol to increase the myocardial expression of GATA-4 in DNR rats is owing to its antiapoptotic properties.

OPN, a key component of the extracellular matrix, is associated with the fibrotic process during tissue remodeling [30]. Recent studies indicate that OPN expression is obligatory for the formation of pathological myocardial fibrosis in rodents [31] and strongly associated with LV hypertrophy [32]. However, the role of OPN in anthracycline-induced cardiomyopathy has not been elucidated. In this study, we have shown that OPN myocardial expression was enhanced in DNR rats compared with that in control group. It is of interest that, in the present study, carvedilol could suppress the enhanced expression of OPN in DNR rats along with decreased myocyte size (Figure 1(b)) and area of myocardial fibrosis (Figures 1(c) and 1(d)). We hypothesize that the increased expression of OPN in this animal model is due to the oxidative stress, since there is cross-talk between the level of OPN and oxidative stress in patients with heart diseases [33]. In addition, the effect of carvedilol on the prevention of fibrosis and hypertrophy might partially be mediated through the inhibition of OPN expression. However, this hypothesis is still speculative, and further evidence is necessary to support it. 

Our earlier reports showed that carvedilol reduced the cardiac fibrosis and inhibited the progression of heart failure in rats with dilated cardiomyopathy [17, 34]. Previous studies reported that carvedilol could prevent myocardial fibrosis in hamster models of progressive cardiomyopathy [35]. It is well established that TGF-*β*1 is a fibrogenic cytokine which is an important modulator in the ventricular remodeling process [36], and carvedilol treatment reduced the expression of TGF-*β*1 [37]. Similar with those above results, in this study, cotreatment with carvedilol also led to the reduction in myocardial fibrosis as evident from decreased area of myocardial fibrosis with Azan-Mallory staining (Figure 1(c)) and reduced myocardial expression of TGF-*β*1 (Figures 2(a) and 2(d)). These results may account for the preventive effects of carvedilol on DNR-induced cardiomyopathy and propose a new effect of this drug on suppression of TGF-*β*1.

**Figure 3:**
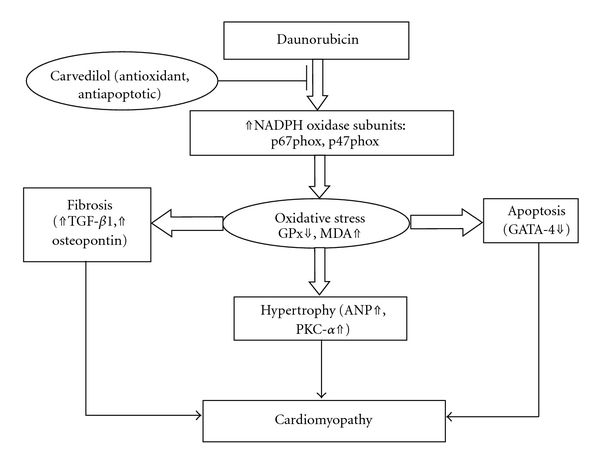
The possible mechanism by which carvedilol afforded protection against DNR-induced cardiomyopathy.

In conclusion, the present study indicates that carvedilol increases survival rate, prevents systolic and diastolic dysfunction, and attenuates myocardial fibrosis and hypertrophy in DNR-induced cardiomyopathic rats. The possible mechanism (Figure 3) by which carvedilol can reduce fibrosis in DNR rats is decreasing oxidative stress-related factors and further decreasing the levels of TGF-*β*1 and OPN. In addition, carvedilol inhibited hypertrophy (as shown by decreased myocardial ANP and PKC-*α* levels) possibly via its antioxidant properties. Moreover, carvedilol also attenuated the reduction in GATA-4 level and prevented myocardial apoptosis. All these findings might explain the ability of carvedilol to prevent cardiomyopathy induced by DNR. The results of the present study add the available evidences on the cardioprotective action of carvedilol when associated with anthracyclines and explain the mechanisms underlying the benefits of their coadministration.
